# The assessment of postoperative cholangitis in malignant biliary obstruction: a real-world study of nasobiliary drainage after endoscopic placement of self-expandable metal stent

**DOI:** 10.3389/fonc.2024.1440131

**Published:** 2024-11-14

**Authors:** Hengwei Jin, Chang Fu, Xu Sun, Changqing Fan, Junhong Chen, Hao Zhou, Kai Liu, Hongji Xu

**Affiliations:** ^1^ Department of Hepatobiliary and Pancreatic Surgery, General Surgery Center, The First Hospital of Jilin University, Changchun, Jilin, China; ^2^ Clinical Medical College, Changchun University of Traditional Chinese Medicine, Changchun, Jilin, China; ^3^ Department of Abdominal Surgery, Guiqian International General Hospital, Guiyang, Guizhou, China

**Keywords:** cholestasis, endoscopic metallic biliary endoprosthesis, endoscopic nasobiliary drainage, cholangitis, recovery

## Abstract

**Objectives:**

Endoscopic retrograde cholangiopancreatography(ERCP) with endoscopic metallic biliary endoprosthesis(EMBE) serves as a crucial palliative treatment for advanced malignant biliary obstruction(MBO). While endoscopic nasobiliary drainage(ENBD) effectively reduces post-ERCP cholangitis (PEC) incidence, its impact on PEC in MBO patients is unclear. This study evaluates ENBD’s effects on PEC in patients undergoing EMBE and identifies risk factors.

**Methods:**

This retrospective cohort study at the First Hospital of Jilin University involved MBO patients who underwent EMBE from September 2011 to September 2022. Propensity score matching (PSM) was applied to minimize selection bias. Primary and secondary outcomes included the incidence and recovery rate/time of PEC, biliary drainage success, and hospitalization duration. Univariate, multivariate, and Lasso regression analyses identified independent risk factors.

**Results:**

In this study of 1,008 patients, 730 were analyzed after PSM(365 each in the EMBE+ENBD and EMBE groups). No significant differences were observed in PEC incidence(10.7% vs 11.2%, p=0.9057) or recovery rates(48.7% vs 31.7%, p=0.1855). However, PEC recovery time was shorter in the EMBE+ENBD group(4.0 days [3.0, 6.0] vs 5.0 days [4.0, 7.5], p=0.0240), as was hospitalization duration(6.0 days [4.0, 8.0] vs 7.0 days [5.0, 10.0], p=0.0146), and a higher success rate of biliary drainage(54.0% vs 43.3%, p=0.0049). Tumor location(HR 1.10, 95% CI 1.00-1.20) and preoperative total bilirubin(HR 2.13, 95% CI 1.66-2.73) were identified as independent risk factors.

**Conclusion:**

In this large-scale PSM study, ENBD did not reduce PEC incidence but expedited recovery and shortened hospital stays. Patients with hilar MBO of Bismuth III-IV or high preoperative bilirubin were more prone to PEC.

## Introduction

Malignant biliary obstruction (MBO) is caused by the invasion or compression of bile ducts by tumors such as cholangiocarcinoma, pancreatic carcinoma, gallbladder carcinoma, ampullary carcinoma, and metastatic diseases (1). Due to nonspecific early symptoms, MBO typically presents at advanced stages, with fewer than 20% of patients being candidates for curative resection (2). To relieve obstruction and prolong survival, Endoscopic Retrograde Cholangiopancreatography (ERCP) is commonly performed for biliary drainage (3, 4). Compared to plastic stents used in endoscopic retrograde biliary drainage (ERBD), endoscopic metallic biliary endoprosthesis (EMBE) with self-expandable metal stents offers longer patency (5), lower reintervention rates (6), and reduced risk of stent migration (5, 7), thereby improving quality of life.

Cholangitis, a common adverse event post-ERCP (PEC), occurs in 1% to 3% of cases (3, 8). Risk factors include invasive procedures like cholangiography (3) and cholangioscopy (9), which may cause bacterial infections, inadequate biliary drainage, and a history of primary sclerosing cholangitis (PSC) (10). In patients with MBO undergoing EMBE, PEC incidence ranges from 6% to 25.9% (6, 11–13), with poor general health and inadequate biliary drainage in hilar MBO patients being significant risk factors (10, 14).

Multiple guidelines state that endoscopic nasobiliary drainage (ENBD) reduces biliary pressure, minimizes intestinal reflux, and lowers the risk of PEC (3, 4, 15). However, there is no consensus on postoperative nasobiliary tube placement in MBO patients undergoing EMBE, and the impact of ENBD on PEC remains unclear. Limited clinical studies exist on the short-term benefits of combining EMBE with ENBD for MBO treatment (16). Thus, we designed a real-world study to retrospectively analyze the impact of ENBD on PEC in MBO patients with self-expandable metal stents.

## Methods

### Study design and study population

Consecutive patients diagnosed with hepatobiliary cancer at the First Hospital of Jilin University from September 2011 to September 2022 were included in this study. Diagnoses followed the National Comprehensive Cancer Network Clinical Practice Guidelines for Hepatobiliary Cancers-Evidence Blocks (1). Patient treatments were determined through multidisciplinary discussions.

Inclusion criteria: a)Patients with diagnosed MBO, unsuitable for curative resection due to metastasis or poor health;b) Patients with symptoms of significant biliary obstruction (jaundice, abdominal pain, anorexia);c) Age≥18 years;d) Patients receiving the first ERCP and self-expandable metal stent. Exclusion criteria: a) Patients with concomitant cholelithiasis or choledocholithiasis;b) Patients who have undergone percutaneous transhepatic cholangial drainage (PTCD);c) Patients with preoperative acute cholangitis.

Patients were categorized into the EMBE group and the EMBE + ENBD group based on whether ENBD was performed. Clinical data were extracted from the Real World Data Platform of the First Hospital of Jilin University. Baseline characteristics and relevant variables were collected using clinical expertise and literature evidence (3, 8–10, 17, 18). This study received approval from the Ethics Committee of the First Hospital of Jilin University (2024-006) and was conducted according to the Declaration of Helsinki.

### ERCP procedure

**Figure 1:**
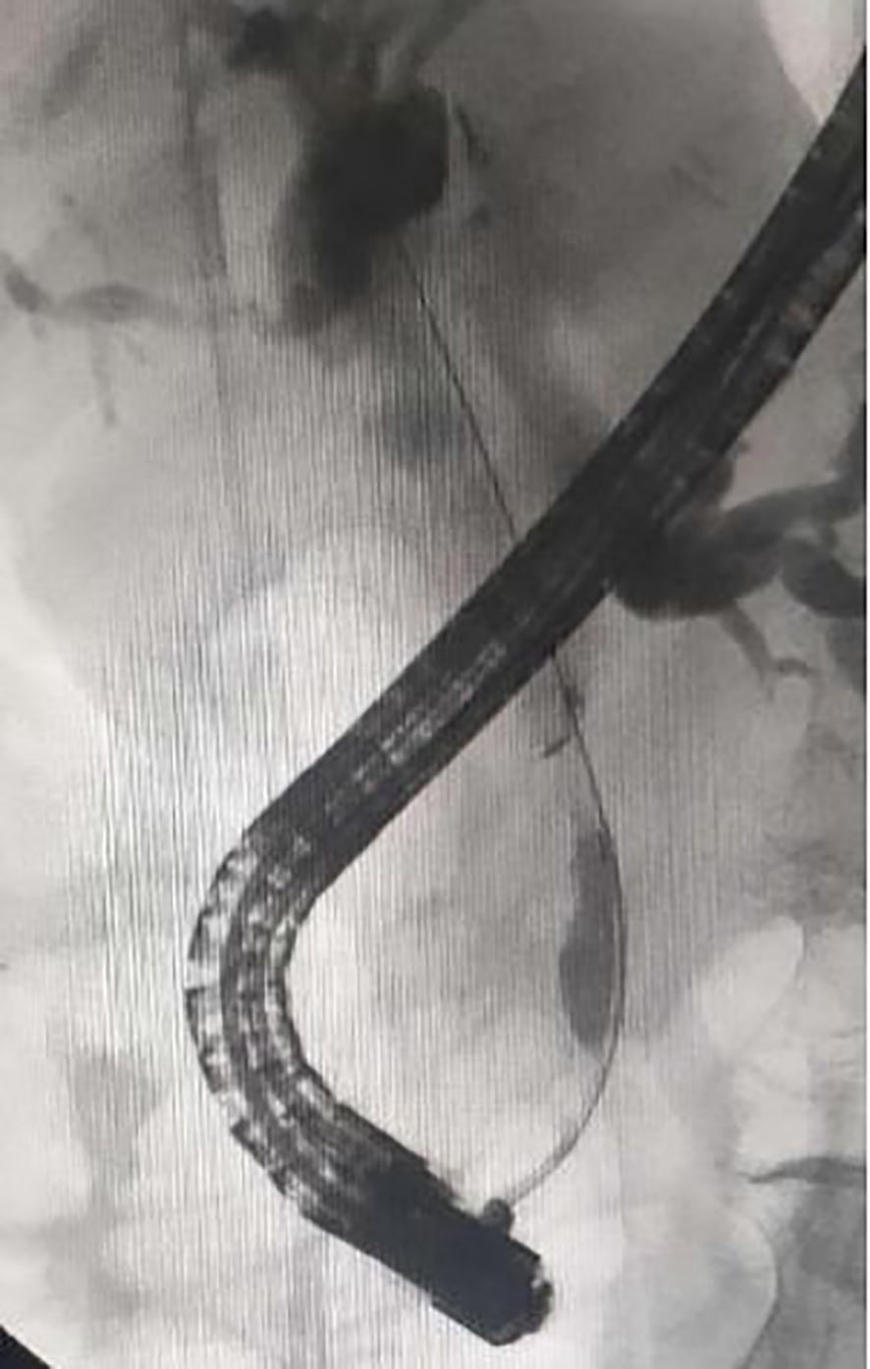
Cholangiography showing malignant biliary obstruction involving in the middle of common bile duct.

**Figure 2:**
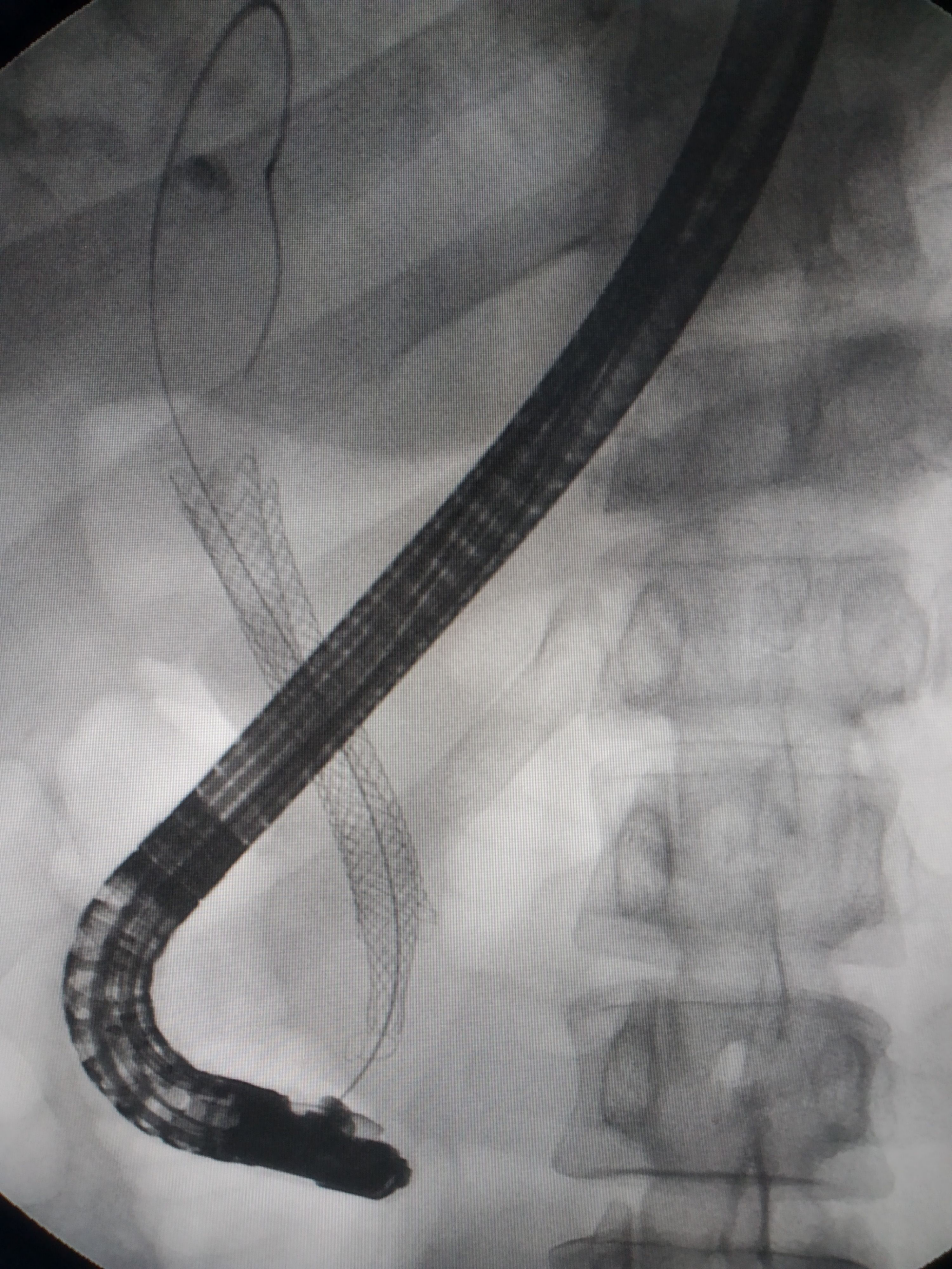
Cholangiography showing the self-expandable metal stent across the stricture.

**Figure 3:**
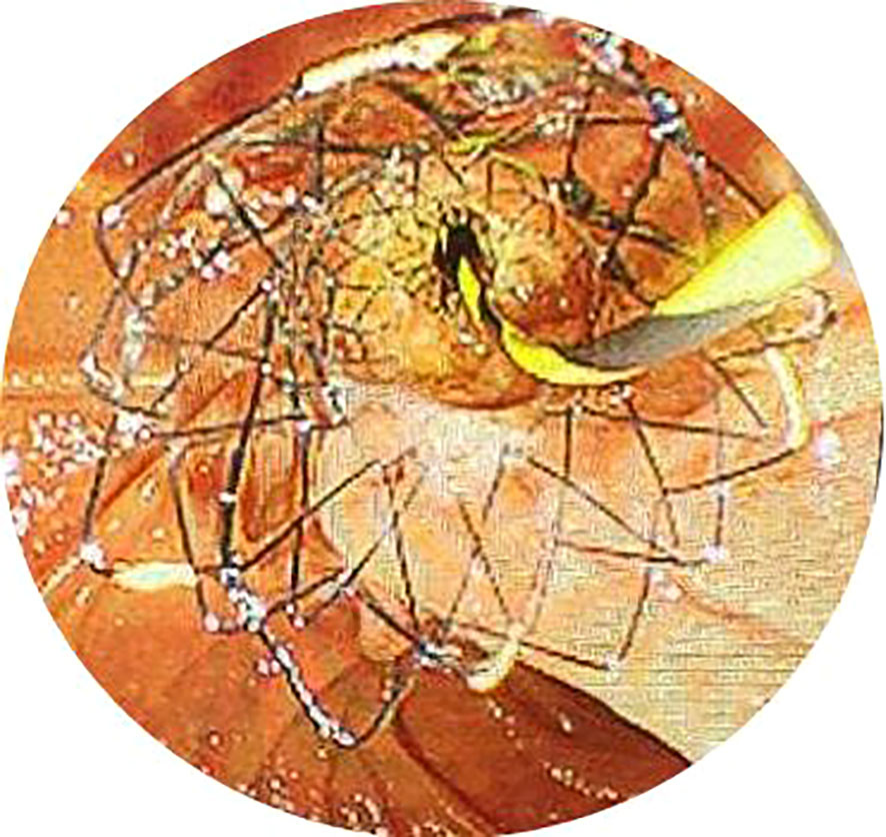
The metal stent was placed in the bile duct with the end located in the intestine under endoscopy.

**Figure 4:**
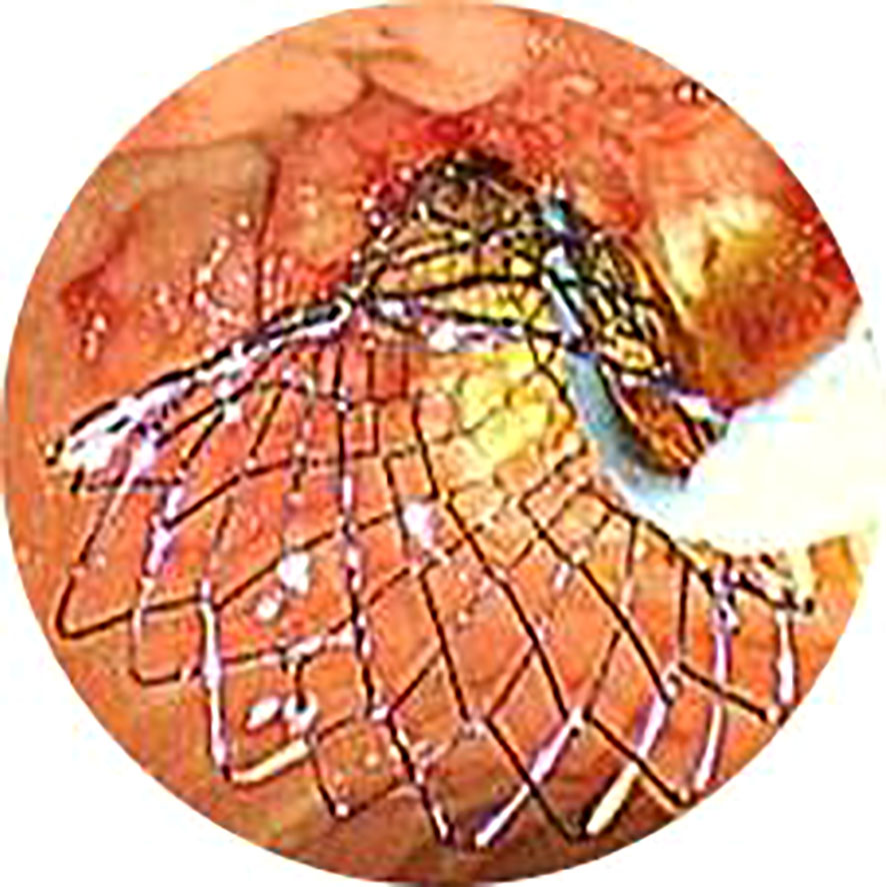
In the EMBE+ENBD group, one nasobiliary catheter with negative pressure drainage device was deployed after insertion of metal stents. *EMBE, endoscopic metallic biliary endoprosthesis; ENBD, endoscopic nasobiliary drainage*.

After obtaining ERCP informed consent, all patients received prophylactic antibiotics and non-steroidal anti-inflammatory drugs preoperatively. Procedures were performed under conscious sedation or general anesthesia by skilled endoscopists. ERCP was conducted in the prone position using a standard therapeutic duodenoscope (TJF-260V, Olympus, Tokyo, Japan). Successful cannulation of the common bile duct led to cholangiography, which delineated the location and extent of biliary strictures (**Figure 1**). For difficult cannulations, pre-cut sphincterotomy, endoscopic sphincterotomy (EST), or endoscopic papillary balloon dilation (EPBD) were employed (**Supplementary Materials Figures 1**, **2**). Endoscopic radiofrequency ablation (RFA) was performed as needed (**Supplementary Materials Figures 3**, **4**). Endoscopic retrograde pancreatic drainage (ERPD) was considered to prevent post-ERCP pancreatitis following pancreatic duct cannulation (**Supplementary Materials Figure 5**). Metal stents were optimally positioned under fluoroscopic guidance (**Figures 2**, **3**). In the EMBE + ENBD group, a nasobiliary tube with a negative pressure device was inserted through the stenosis(**Figure 4**). Broad-spectrum antibiotics were administered postoperatively, with regular monitoring of blood biochemical markers, including liver and kidney functions. Nasobiliary tubes were removed after 24 hours if no PEC occurred.

### Definition of events

Primary Outcome: PEC is defined as the presence of one item from group a, one from group d, and one from either group b or c (19). a)Systemic Inflammation after ERCP (a-1: Fever(body temperature> 38°C) and/or shaking chills;a-2: Laboratory data showing evidence of inflammatory response(white blood cell(WBC): < 4*10^9^/L or > 10 *10^9^/L)); b)Cholestasis (b-1: Jaundice(total bilirubin≥34.2µmol/L);b-2: Abnormal liver function tests (ALP, GGT, AST or ALT: > 1.5 times the standard deviation of the normal range)); c) Imaging evaluation(c-1: Biliary dilatation; c-2: Evidence of the etiology on imaging); d)Exclusion of post-ERCP pancreatitis and other infections.

Secondary Outcomes: Biliary drainage success (20) is defined as a return to normal or a decrease of over 25% in total bilirubin levels within two weeks. Hospitalization duration is the number of days from admission to discharge. PEC recovery is achieved when successful biliary drainage coincides with the normalization of clinical symptoms and inflammatory markers post-treatment (19). The observation period for evaluating biliary drainage is also set at two weeks.

Adverse events: Adverse events are classified according to the American Society for Gastrointestinal Endoscopy (ASGE) lexicon for endoscopic adverse events (21), including post-ERCP pancreatitis, bleeding, perforation and death.

### Sample size

A study by Xinjian Wan et al. involving 378 patients reported PEC rates of 11.9% in the EMBE group and 2.4% in the EMBE+ENBD group (16), suggesting that combining EMBE with ENBD could reduce incidence rates. Literature review showed the lowest PEC incidence after EMBE was 6% (6, 11–13). Since this rate did not distinguish between cases with or without ENBD, it may underestimate PEC incidence in the EMBE group, aiding in a more moderate sample size calculation. This study assumes a PEC incidence of 6% for EMBE and 2.4% for EMBE+ENBD. Targeting an 80% statistical power and a 0.1 type I error (one-sided), 560 cases are needed (280 per group), adjusted to 672 cases (336 per group) to account for a 20% dropout rate.

### Statistical analysis

Statistical analysis was conducted using R version 4.1.1. P values of less than 0.05 were considered significant. Results were summarized by frequency and percentage for categorical variables, means and standard deviations for normally distributed continuous variables, and medians with interquartile ranges for skewed continuous variables. Chi-square or Fisher’s exact tests were used for categorical variables, while t-tests or Mann-Whitney U tests were used for continuous variables, depending on their distribution. The Kaplan-Meier method and log-rank tests analyzed PEC recovery time. Propensity score matching (PSM) minimized selection bias using the “MatchIt” R package, with scores calculated from a logistic model including six predictors: EPBD, RFA, operative duration, type of cancer, preoperative AST, and TB. Predictors were selected based on baseline imbalances and literature support. Patients were matched using the nearest neighbor method with a caliper width of 0.1, without replacement.

Univariate and multivariate analyses were conducted on the pre-matching dataset to assess risk factors for PEC. Univariate logistic regression identified clinical variables impacting outcomes using the “autoReg” R package. Variables with p-values < 0.1 were included in the multivariate logistic regression; those statistically significant were deemed independent risk factors. Lasso regression was employed to validate the multivariate analysis results using the “glmnet” R package.

## Results

### Baseline characteristics of MBO patients that received EMBE

**Figure 5:**
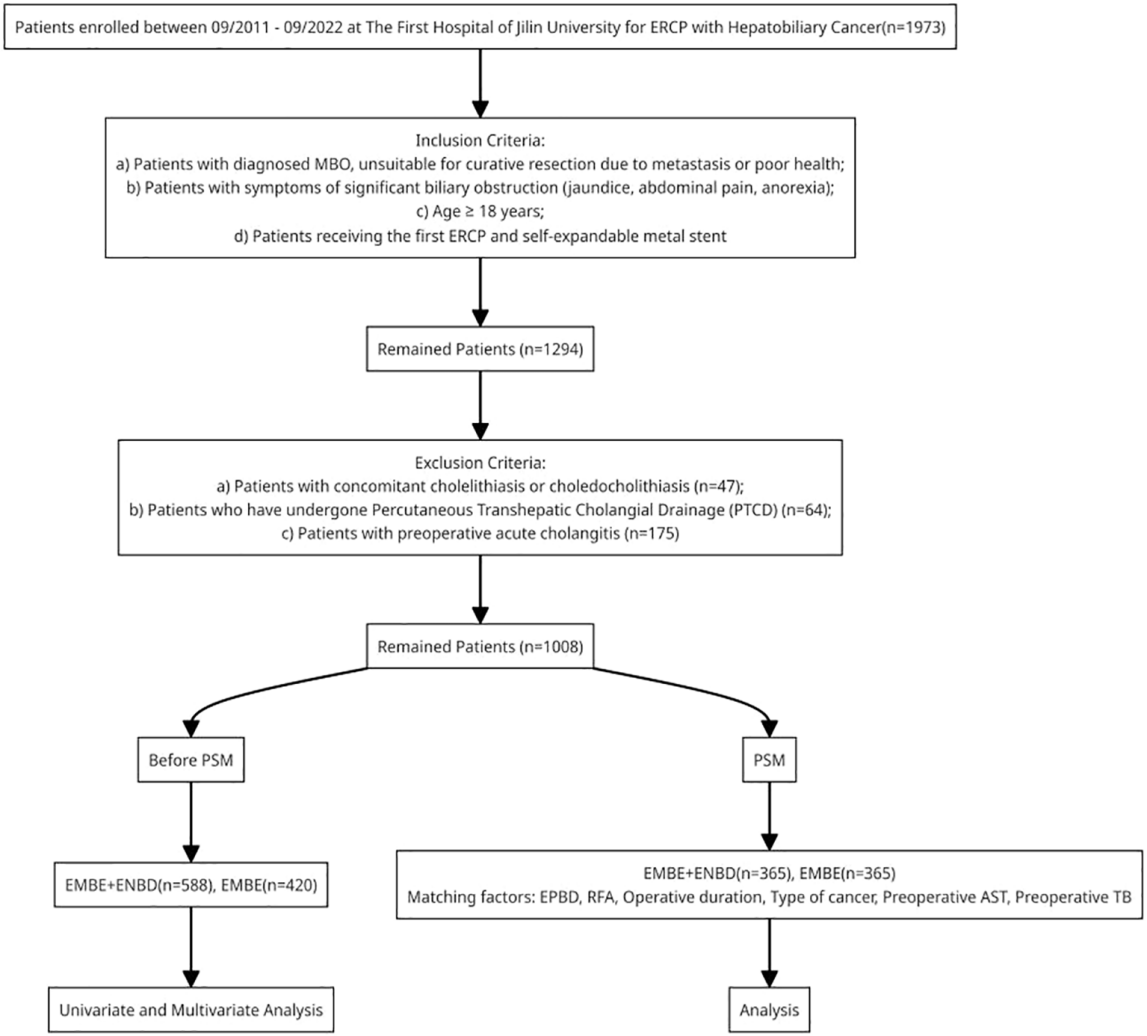
Flowchart of patients inclusion. MBO, malignant biliary obstruction; PSM, propensity score matching; ERCP, endoscopic retrograde cholangiopancreatography; EMBE, endoscopic metallic biliary endoprosthesis; ENBD, endoscopic nasobiliary drainage.

From September 2011 to September 2022, 1,973 MBO patients with hepatobiliary cancer were admitted to our hospital. Among them, 1,008 were enrolled in the study: 420 (41.7%) in the EMBE group and 588 (58.3%) in the EMBE+ENBD group (**Figure 5**). Demographic and clinical characteristics are shown in **Table 1**. The median age was 67 years [59.0, 74.0], with 619 males (61.4%). The most frequent MBO causes were cholangiocarcinoma (637, 63.2%) and pancreatic carcinoma (297, 29.5%). Obstruction locations were Bismuth I-II (180, 17.9%), Bismuth III-IV (281, 27.9%), and non-hilar (547, 54.3%). Interventions included EST (855, 84.8%), EPBD (895, 88.8%), ERPD (287, 28.5%), and RFA (34, 3.4%), with a median operative duration of 0.7 hours [0.4, 1.1].

**Table 1:** Baseline demographic and clinical characteristics of the patients.

	Main cohort				Matched cohort		
	Overall (n=1008)	EMBE (n=420)	EMBE+ENBD (n=588)	P value	EMBE (n=365)	EMBE+ENBD (n=365)	P value
**Age, year**	67.0 [59.0,74.0]	67.0[59.0,73.0]	67.0[60.0,74.2]	0.196	67.0 [59.0, 73.0]	67.0 [59.0, 75.0]	0.536
**Sex**				0.939			0.820
Male	619 (61.4%)	259 (61.7%)	360 (61.2%)		224 (61.4%)	220 (60.3%)	
Female	389 (38.6%)	161 (38.3%)	228 (38.8%)		141 (38.6%)	145 (39.7%)	
**Race**				0.241			0.800
Han	914 (90.7%)	375 (89.3%)	539 (91.7%)		329 (90.1%)	332 (91.0%)	
Others	94 (9.3%)	45 (10.7%)	49 (8.3%)		36 (9.9%)	33 (9.0%)	
**BMI, kg/m^2^**	22.5 [20.0, 24.9]	22.5 [20.1, 25.0]	22.5 [20.0, 24.9]	0.925	22.4 [20.0, 25.1]	22.5 [20.0, 24.8]	0.782
**Type of cancer**				<0.001			0.126
Cholangiocarcinoma	637 (63.2%)	247 (58.8%)	390 (66.3%)		227 (62.2%)	225 (61.6%)	
Pancreatic	297 (29.5%)	137 (32.6%)	160 (27.2%)		118 (32.3%)	114 (31.2%)	
Gallbladder	23 (2.3%)	4 (1.0%)	19 (3.2%)		3 (0.8%)	13 (3.6%)	
Ampullary	32 (3.2%)	20 (4.8%)	12 (2.0%)		10 (2.7%)	9 (2.5%)	
Hepatocellular carcinoma	19 (1.9%)	12 (2.9%)	7 (1.2%)		7 (1.9%)	4 (1.1%)	
**Tumor location**				0.026			0.975
Hilar Bismuth I-II	180 (17.9%)	66 (15.7%)	114 (19.4%)		64 (17.5%)	65 (17.8%)	
Hilar Bismuth III-IV	281 (27.9%)	105 (25.0%)	176 (29.9%)		97 (26.6%)	99 (27.1%)	
Non-hilar	547(54.3%)	249(59.3%)	298(50.7%)		204 (55.9%)	201 (55.1%)	
Tumor marker before ERCP							
CEA,ng/mL	4.4 [2.4, 18.0]	4.5 [2.5, 18.0]	4.4 [2.4, 18.0]	0.674	4.5 [2.6, 18.0]	4.4 [2.5, 18.0]	0.990
AFP,ng/mL	4.0 [2.7, 9.9]	4.3 [2.8, 25.9]	3.7 [2.6, 6.6]	<0.001	4.3 [2.8, 25.9]	3.7 [2.7, 7.4]	0.071
CA125,U/mL	31.7 [15.6, 68.5]	38.1 [16.6, 68.5]	27.0 [15.0, 68.5]	0.060	37.4 [16.6, 68.5]	29.5 [14.9, 68.5]	0.133
CA19-9,U/mL	374.4 [139.1, 668.0]	374.4 [162.1, 577.4]	374.4 [124.2, 740.3]	0.921	374.4 [170.2, 657.4]	374.4 [122.6, 717.9]	0.603
Blood test before ERCP							
WBC,10^9^/L	6.5 [5.2, 8.0]	6.3 [5.1, 7.8]	6.7 [5.2, 8.1]	0.068	6.4 [5.1, 7.9]	6.7 [5.1, 8.1]	0.376
NC (%)	0.8 [0.7, 0.8]	0.8 [0.7, 0.8]	0.8 [0.7, 0.8]	0.201	0.8 [0.7, 0.8]	0.8 [0.7, 0.8]	0.764
Blood platelet,10^9^/L	206.0 [158.0, 267.0]	206.5 [157.0, 270.0]	206.0 [159.0, 265.0]	0.879	206.0 [160.0, 269.0]	207.0 [159.0, 264.0]	0.949
Hemoglobin,g/L	113.0 [99.8, 125.0]	113.0 [99.0, 126.0]	113.0 [100.0, 124.0]	0.941	113.0 [99.0, 126.0]	112.0 [99.0, 122.0]	0.553
Biochemical indicator before ERCP							
Creatinine,µmol/L	58.0 [48.1, 69.3]	57.8 [46.9, 71.2]	58.1 [48.7, 68.3]	0.665	57.8 [47.1, 70.2]	58.3 [48.5, 67.4]	0.815
GGT,U/L	536.7 [260.3, 898.9]	437.4 [203.1, 836.9]	585.9 [303.1, 935.3]	<0.001	448.6 [207.5, 852.5]	580.8 [284.9, 898.9]	0.128
AST,U/L	85.0 [50.3, 147.7]	76.3 [45.3, 139.5]	87.8 [54.8, 156.5]	<0.001	82.7 [47.4, 143.9]	84.2 [53.4, 145.7]	0.188
ALT,U/L	104.0 [52.8, 192.1]	97.8 [48.2, 186.6]	108.7 [58.6, 195.8]	0.057	101.6 [51.6, 192.0]	104.0 [55.9, 192.7]	0.843
TB,µmol/L	165.6 [85.4, 287.1]	148.8 [79.2, 267.3]	182.6 [95.2, 301.6]	0.002	155.9 [82.6, 279.3]	174.9 [97.7, 278.4]	0.161
**Body temperature before** **ERCP,°C**	36.5 [36.5, 36.6]	36.5 [36.5, 36.6]	36.5 [36.5, 36.6]	0.765	36.5 [36.5, 36.6]	36.5 [36.5, 36.6]	0.167
**EST**				0.168			0.244
Yes	855 (84.8%)	348 (82.9%)	507 (86.2%)		319 (87.4%)	307 (84.1%)	
No	153 (15.2%)	72 (17.1%)	81 (13.8%)		46 (12.6%)	58 (15.9%)	
**EPBD**				<0.001			0.795
Yes	895 (88.8%)	343 (81.7%)	552 (93.9%)		331 (90.7%)	334 (91.5%)	
No	113 (11.2%)	77 (18.3%)	36 (6.1%)		34 (9.3%)	31 (8.5%)	
**ERPD**				1.00			0.749
Yes	287 (28.5%)	120 (28.6%)	167 (28.4%)		111 (30.4%)	116 (31.8%)	
No	721 (71.5%)	300 (71.4%)	421 (71.6%)		254 (69.6%)	249 (68.2%)	
**RFA**				<0.001			1.00
Yes	34 (3.4%)	3 (0.7%)	31 (5.3%)		3 (0.8%)	3 (0.8%)	
No	936 (92.9%)	395 (94.0%)	541 (92.0%)		362 (99.2%)	362 (99.2%)	
**Operative duration,hour**	0.7 [0.4, 1.1]	0.6 [0.4, 1.0]	0.8 [0.5, 1.2]	<0.001	0.6 [0.4, 1.0]	0.8 [0.5, 1.0]	0.091

Values are mean ± standard deviation,medians with interquartile ranges or n (%).

BMI, body mass index; WBC, white blood cell;NC, neutrophils count;GGT, gamma-glutamyl transpeptidase; AST, aspartate amino transferase ;ALT, alanine amino transferase;TB, total bilirubin;EST, endoscopic sphincterotomy;EPBD, endoscopic papillary balloon dilation;ERPD,endoscopic retrograde pancreatic drainage;RFA,radiofrequency ablation; EMBE,endoscopic metallic biliary endoprosthesis; ENBD,endoscopic nasobiliary drainage.

### Propensity score matching

Statistical differences were observed between the EMBE and EMBE+ENBD groups in nine factors: type of cancer (p<0.001), tumor location (p=0.026), preoperative AFP (p<0.001), gamma-glutamyl transpeptidase (p<0.001), aspartate amino transferase (p<0.001), TB (p=0.002), EPBD (p<0.001), RFA (p<0.001), and operative duration (p<0.001). Based on literature (3, 8–10, 17) and expert opinions, six factors were selected for PSM: EPBD, RFA, operative duration, type of cancer, preoperative AST, and TB. After PSM, there were 365 patients in each group, showing no significant differences in baseline characteristics (**Table 1**). We assessed PSM effectiveness by plotting density graphs for each covariate before and after matching (R package “cobalt”). Results showed that matching made the factors more consistent between groups, enhancing the study’s methodological quality and reliability (**Supplementary Materials Figure 6**).

### Outcomes of the cohort after PSM

**Figure 6:**
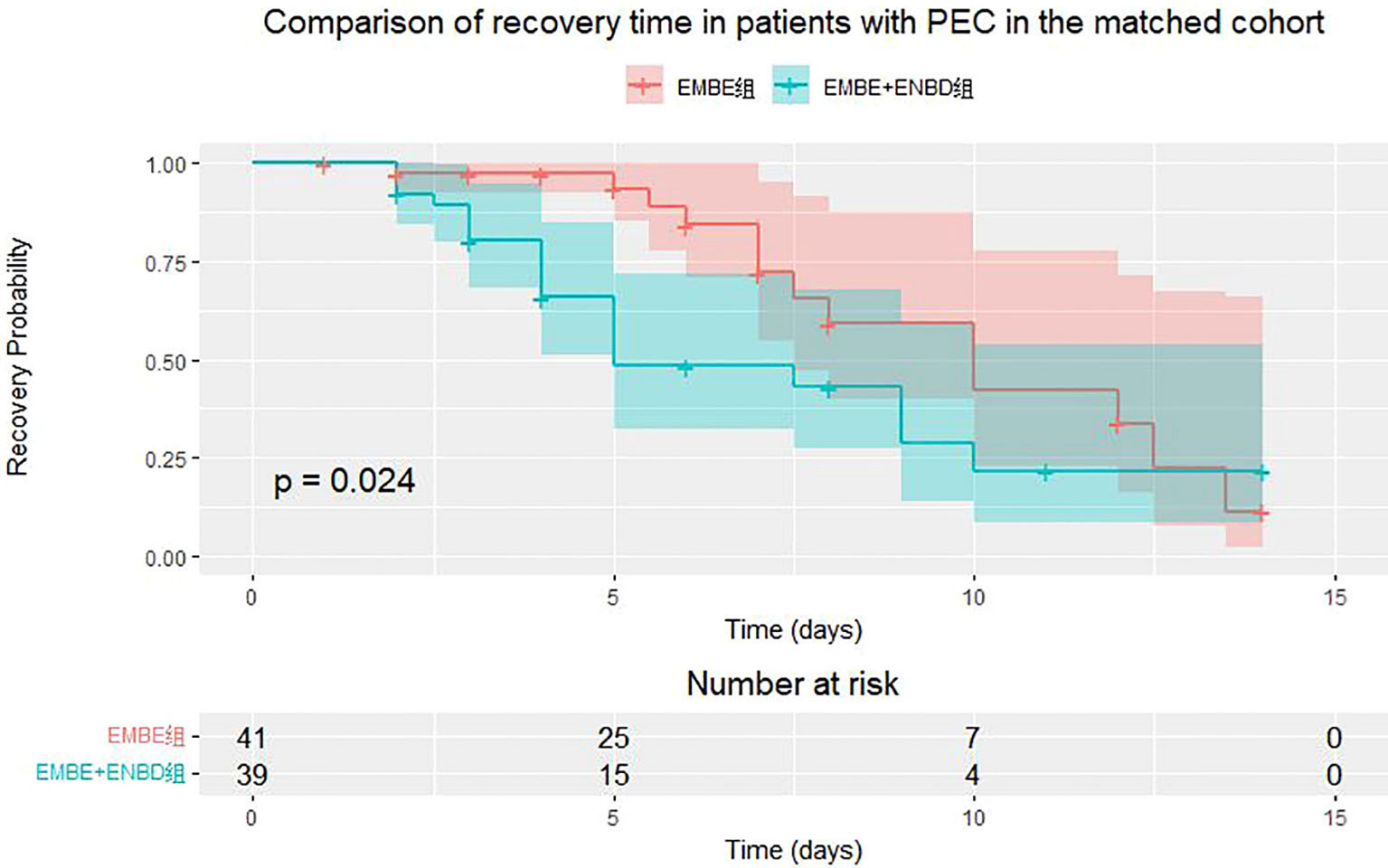
Kaplan-Meier curve for post-ERCP cholangitis (PEC) recovery time in EMBE+ENBD and EMBE groups.

In the matched cohort of 730 patients, the incidence of PEC was similar between the EMBE+ENBD group (39, 10.7%) and the EMBE group (41, 11.2%), showing no significant difference (p=0.9057) (**Table 2**). Over a 2-week observation period, PEC recovery rates showed no statistical difference (19 (48.7%) vs. 13 (31.7%), p=0.1855). PEC recovery time was documented. If outcomes were not observed within the observation period due to discharge or other reasons, these were noted and categorized as lost to follow-up. Median recovery times were 4.0 days [3.0, 6.0] for EMBE+ENBD and 5.0 days [4.0, 7.5] for EMBE, showing a significant difference (p=0.0240, log-rank test, **Figure 6**). Additionally, biliary drainage success (197 (54.0%) vs. 158 (43.3%), p=0.0049) and hospitalization duration (6.0 days [4.0, 8.0] vs. 7.0 days [5.0, 10.0], p=0.0146) were superior in the EMBE+ENBD group.

**Table 2:** Outcomes of EMBE and EMBE + ENBD group in the matched cohort.

	Matched cohort			
	Overall (n=730)	EMBE (n=365)	EMBE+ENBD (n=365)	P value
**Post-ERCP cholangitis**	80 (11.0%)	41 (11.2%)	39 (10.7%)	0.9057
Post-ERCP cholangitis recovery	32 (40.0%)	13 (31.7%)	19 (48.7%)	0.1855
Post-ERCP cholangitis recovery time,day	4.5 [3.0, 7.1]	5.0 [4.0, 7.5]	4.0 [3.0, 6.0]	0.0240^*^
**Biliary drainage success**	355 (48.6%)	158 (43.3%)	197 (54.0%)	0.0049
**Hospitalization duration,day**	7.0 [5.0, 10.0]	7.0 [5.0, 10.0]	6.0 [4.0, 8.0]	0.0146

Values are mean ± standard deviation,medians with interquartile ranges or n (%).

EMBE, endoscopic metallic biliary endoprosthesis; ENBD, endoscopic nasobiliary drainage; ERCP, endoscopic retrograde cholangiopancreatography.

*Log-rank test.

Regarding ERCP-related adverse events (**Table 3**), the post-ERCP pancreatitis rate was 3.3% overall (2.5% EMBE+ENBD vs. 4.1% EMBE, p=0.2994). Bleeding was similar at 1.9% for EMBE+ENBD and 2.2% for EMBE (p=1.0000). Perforation occurred in 0.5% of EMBE+ENBD and 0.3% of EMBE patients (p=1.0000). There was no significant difference in mortality between the groups, each at 0.3% (p=1.0000). Similar adverse event outcomes were noted in the cohort before matching.

**Table 3:** Adverse events of EMBE and EMBE + ENBD group.

	Main cohort				Matched cohort			
	Overall (n=1008)	EMBE (n=420)	EMBE+ENBD (n=588)	P value	Overall (n=730)	EMBE (n=365)	EMBE+ENBD (n=365)	P value
**Post-ERCP pancreatitis**	33 (3.3%)	15 (3.6%)	18 (3.0%)	0.7877	24 (3.3%)	15 (4.1%)	9 (2.5%)	0.2994
**Bleeding**	19 (1.9%)	11 (2.6%)	8 (1.4%)	0.2249	15 (2.1%)	8 (2.2%)	7 (1.9%)	1.0000
**Perforation**	6 (0.6%)	3 (0.7%)	3 (0.5%)	0.6978^*^	3 (0.4%)	1 (0.3%)	2 (0.5%)	1.0000^*^
**Death**	3 (0.3%)	2 (0.5%)	1 (0.2%)	0.5741^*^	2 (0.3%)	1 (0.3%)	1 (0.3%)	1.0000^*^

Values are n (%).

EMBE, endoscopic metallic biliary endoprosthesis; ENBD, endoscopic nasobiliary drainage; ERCP, endoscopic retrograde cholangiopancreatography.

*Fisher’s Exact Test.

### Risk factors for PEC in the cohort before PSM

Based on references and clinical practice, and to prevent multicollinearity, we identified 10 potential risk factors for PEC (**Table 4**). Continuous variables were converted to categorical ones to categorize risk levels. In univariate analysis, significant variables included tumor location (p<0.001), preoperative TB (p<0.001), EPBD (p=0.058), and operative duration (p=0.061). Multivariate analysis revealed significant predictors: tumor location (OR, 1.10; 95% CI, 1.00–1.20; p=0.048) and preoperative TB (OR, 2.13; 95% CI, 1.66–2.73; p<0.001). We calculated the odds ratios for each independent risk factor based on categorical indicators to assess their clinical significance (**Table 5**).

**Table 4:** Univariate and Multivariate analyses for the risk factors for post-ERCP cholangitis.

	Univariate analysis		Multivariate analysis	
	Odds Ratio (95% confidence interval)	P value	Odds Ratio (95% confidence interval)	P value
Age*	0.84 (0.58-1.21)	0.354		
**Type of cancer**	0.80 (0.61-1.06)	0.127		
**Tumor location**	1.15 (1.06-1.26)	<0.001	1.10 (1.00-1.20)	0.048
Preoperative TB*	2.24 (1.76-2.86)	<0.001	2.13 (1.66-2.73)	<0.001
**ENBD**	0.98 (0.65-1.47)	0.931		
**EST**	1.21 (0.67-2.19)	0.523		
**EPBD**	2.27 (0.97-5.30)	0.058	1.68 (0.71-3.98)	0.239
**ERPD**	1.19 (0.77-1.84)	0.424		
**RFA**	1.13 (0.39-3.26)	0.825		
Operative duration*	1.31 (0.99-1.73)	0.061	1.18 (0.87-1.61)	0.297

*Age: Youth: ≤40y, middle age: 40y<age ≤ 65y, senior: >65y.

*Preoperative TB: mild:TB ≤ 171µmol/L,moderate: 171µmol/L<TB ≤ 342µmol/L,severe: >342µmol/L.

*Operative duration: short: <0.4h, middle: 0.4h≤time ≤ 1.0h, long: >1.0h.

TB, total bilirubin;EST, endoscopic sphincterotomy;EPBD, endoscopic papillary balloon dilation;ERPD,endoscopic retrograde pancreatic drainage;RFA,radiofrequency ablation; ENBD, endoscopic nasobiliary drainage; ERCP, endoscopic retrograde cholangiopancreatography.

**Table 5:** The impact of independent risk factor on the post-ERCP cholangitis.

		Z value	Odds Ratio (95% confidence interval)	P value
**Tumor location**	Non-hilar*	–	–	–
	Hilar Bismuth I-II	0.732	1.24 (0.68-2.17)	0.4639
	Hilar Bismuth III-IV	3.219	2.07 (1.33-3.23)	0.0013
**Preoperative TB**	Mild*	–	–	–
	moderate	4.399	3.12 (1.89-5.24)	<0.001
	severe	6.019	5.19 (3.05-8.95)	<0.001

TB, total bilirubin; ERCP, endoscopic retrograde cholangiopancreatography.

Preoperative TB: mild:TB ≤ 171µmol/L,moderate: 171µmol/L<TB ≤ 342µmol/L,severe: >342µmol/L.

*The reference level.

**Figure 7:**
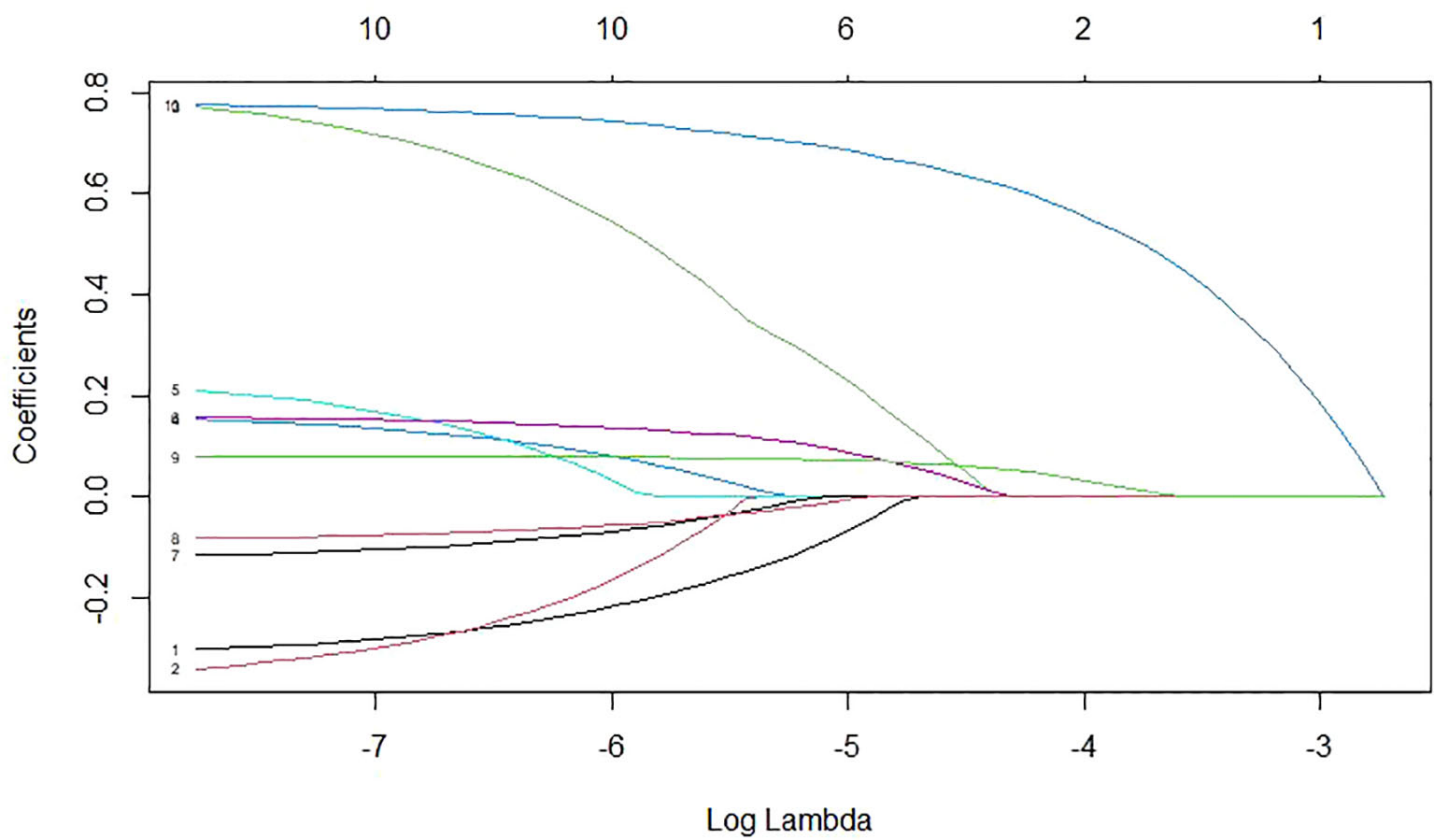
The coefficient variation characteristics of the variables in Lasso regression variable screening.

**Figure 8:**
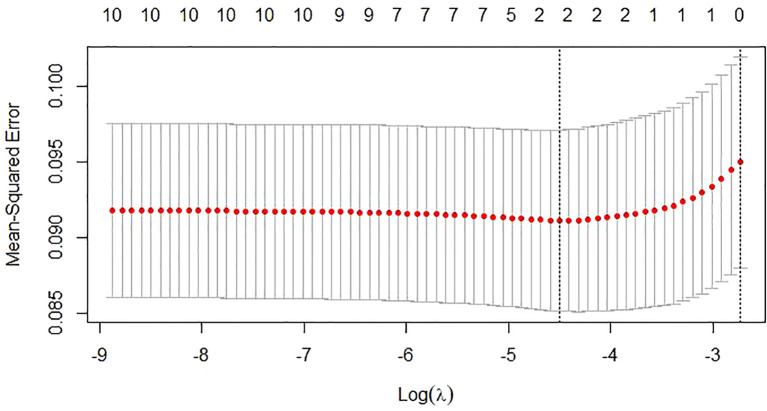
The selection process of the optimum value of the parameter λ in the Lasso regression model by cross-validation.

Risk factors were also screened using Lasso regression, with the coefficient variation characteristics of these variables shown in **Figure 7**. A 10-fold cross-validation method optimized the model to ensure minimal variables and excellent performance, resulting in a λ of 0.01112489 (**Figure 8**). The results of Lasso regression are consistent with those of multivariate analysis, strongly confirming the reliability of the variable selection outcome.

## Discussion

For unresectable MBO, biliary drainage through ERCP is a commonly used and effective treatment (3, 4). EMBE is widely used in clinical practice due to its advantages of prolonged patency (5), low reintervention rates (6), and reduced risk of stent migration (5, 7). PEC is a common postoperative complication that can affect patient recovery, potentially prolong hospital stays and increase medical costs (3, 8, 18). Invasive procedures such as cholangiography (3) and cholangioscopy (9), incomplete biliary drainage, and a history of PSC (10) are recognized risk factors. A retrospective study of 4,324 cases identified hilar obstruction, age ≥60 years, and a history of prior ERCP as independent risk factors, while complete removal of biliary stones was protective (22). Both PSC and hilar obstruction, which cause incomplete biliary drainage, are related to PEC, though no controlled studies are available (10). Another retrospective study of 4,214 ERCPs found that cholangioscopy increased the risk of PEC (9). Some factors are not risk factors for PEC: cirrhosis (23), operator experience (24), and periampullary diverticulum (25).

The mechanisms underlying PEC include bacterial infection and increased biliary pressure (26, 27). The sphincter of Oddi prevents duodenal reflux and ascending bacterial infections. Bile’s flushing action and antibacterial activity of bile salts maintain bile duct sterility. Secretory IgA and biliary mucus act as anti-adhesion factors, preventing bacterial colonization. Invasive procedures introduce bacteria and impair defenses, increasing PEC risk (9). Cholangiography can increase biliary pressure, affecting host defenses, bile flow, and IgA production. This may increase bile duct wall permeability, facilitating bacterial entry into systemic circulation and raising sepsis risk.

Studies indicate that PEC incidence following EMBE can be as high as 6% to 25.9% (6, 11–13). PEC is often reported as an adverse event, with higher incidence rates than conventional ERCP. High-risk factors include poor health and suboptimal biliary drainage in patients with hilar MBO (10, 14). Guidelines suggest that ENBD can reduce PEC occurrence (3, 4, 15), but there’s no consensus on its benefits for EMBE with MBO. Some scholars argue for ENBD to manage temporary biliary obstruction post-EMBE, while others cite increased discomfort and costs (28). Few studies explore this. Xinjian Wan et al’s research shows ENBD is safe and feasible for unresectable hilar MBO, but non-hilar cases may not need ENBD (16). Therefore, it is essential to study the impact of ENBD on the occurrence and recovery of cholangitis post-EMBE to guide clinical practice.

To our knowledge, this is the largest and first retrospective clinical study using PSM to focus on PEC development following EMBE. We evaluated 1008 patients, and after PSM, 730 patients (365 per group) were included in the analysis. The overall incidence of PEC was higher than that following conventional ERCP, with no significant difference between the EMBE and EMBE+ENBD groups. ENBD does not appear to protect against PEC in MBO patients, differing from Wan et al.’s findings. Several reasons may explain this: advanced cancer impairs biliary barrier function and reduces local resistance to bacteria, while EMBE can cause mechanical damage to the bile duct mucosa and increase infection risks (26). Stent expansion might compress local blood vessels, causing ischemia and making the bile duct more susceptible to bacterial attack (17). Tumor thrombi and necrotic tissue can cause temporary stent blockages, leading to poor biliary drainage (8). Hilar MBO may not achieve complete drainage even with stent placement, and procedures like intraoperative cholangiography can increase biliary pressure, contributing to PEC (2, 3). Given these factors, PEC incidence may be higher than with conventional ERCP, and factors causing PEC are present during surgery; optimal biliary drainage with ENBD cannot fully prevent cholangitis. Our study’s large sample size and use of PSM enhance result reliability.

Subsequently, we assessed PEC recovery in both groups based on recovery rate, recovery time, biliary drainage success rate, and hospitalization duration. The EMBE+ENBD group showed shorter PEC recovery time, shorter hospitalization duration, and higher biliary drainage success rates, despite similar recovery rates. This similarity may be due to comparable PEC incidences and aggressive symptomatic treatment. Continuous and unobstructed biliary drainage relieves preoperative biliary obstruction and aids in the recovery of jaundice and PEC. ENBD allows for the assessment of bile quality and quantity, facilitates bacterial culture, and guides antibiotic use, thereby shortening recovery time (29, 30). Adverse event incidence was similar in both groups, indicating that ENBD is safe and does not increase risk.

For the unmatched patient cohort, we analyzed risk factors for PEC. Results indicated that tumor location and preoperative total bilirubin are significant risk factors. Hilar obstructions are more likely to cause cholangitis than distal ones, with Bismuth III-IV obstructions increasing the risk by 107% especially. Hilar MBO affects more bile duct branches, making adequate drainage difficult even after stent placement (5, 14). Surgical manipulations and aggressive tumors increase bacterial infection risk. Moderate and severe jaundice, compared to mild jaundice, increased cholangitis incidence by 212% and 419%. Higher preoperative bilirubin levels, associated with impaired liver function, reduce immune capacity and increase infection risk. Cholestasis enhances bacterial growth, damages mucosal barriers, and facilitates bacterial invasion (31). LASSO regression confirmed these conclusions.

Our study has limitations, including potential biases from its retrospective design and single-center nature. We minimized selection bias through PSM and conducted multivariable analysis to limit confounding factors. However, residual confounding and selection bias may still exist. Further prospective and randomized controlled trials are needed to validate our conclusions.

In conclusion, our large-scale study found no evidence that ENBD after EMBE reduces PEC incidence in MBO patients. However, the EMBE+ENBD group had higher biliary drainage success rates, shorter PEC recovery and hospitalization times, without increased adverse events. Tumor location and preoperative total bilirubin levels were identified as independent risk factors. This suggests that patients with high preoperative bilirubin levels and hilar MBO of Bismuth III-IV are at higher risk for PEC, and nasobiliary tube placement may be beneficial for these patients.

## Data Availability

The raw data supporting the conclusions of this article will be made available by the authors, without undue reservation.
