# Supplementary material for: The assessment of postoperative cholangitis in malignant biliary obstruction: a real-world study of nasobiliary drainage after endoscopic placement of self-expandable metal stent
Source: Front Oncol. 2024 Nov 14;14:1440131. doi: 10.3389/fonc.2024.1440131 (PMC11602394; doi:10.3389/fonc.2024.1440131)
Supplement: Supplementary file 1 [file Image1.pdf]

Supplementary Materials:

Figure 1: Endoscopic sphincterotomy (EST) for difficult cannulations.

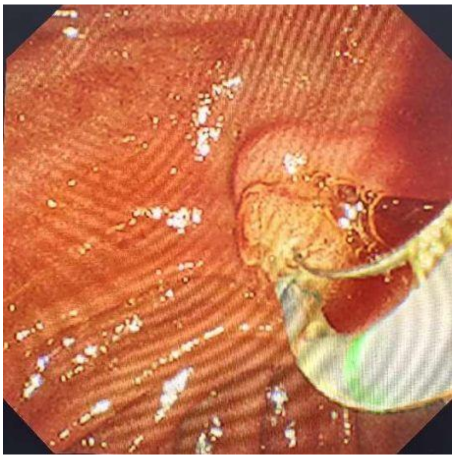

Figure 2: Endoscopic papillary balloon dilation (EPBD) for difficult cannulations.

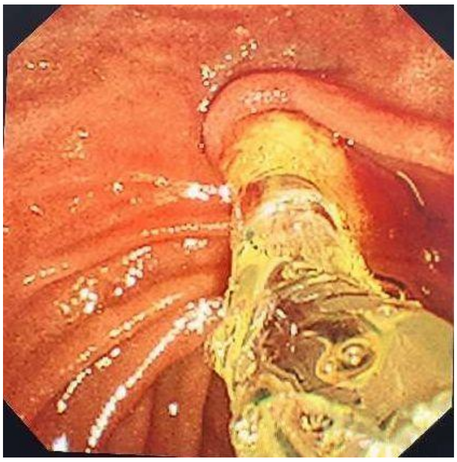

Figure 3: Endoscopic radiofrequency ablation (RFA) is performed when the endoscopists think it's beneficial for the patients.

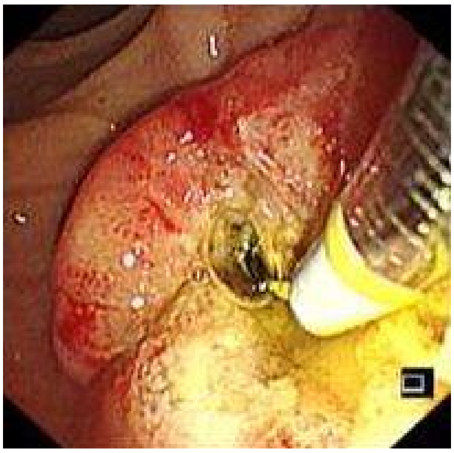

Figure 4: Endoscopic radiofrequency ablation (RFA) is performed under fluoroscopic

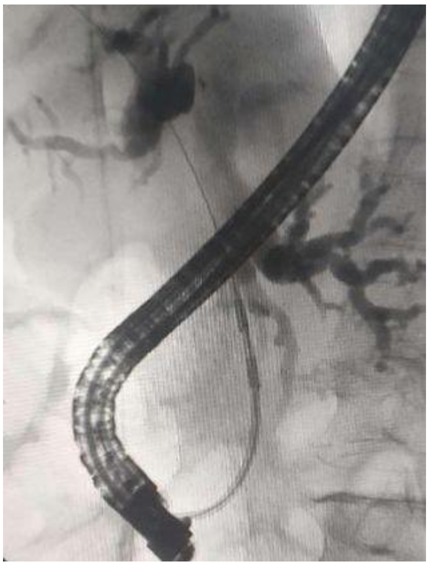

Figure 5: Endoscopic retrograde pancreatic drainage (ERPD) is performed following pancreatic duct cannulation.

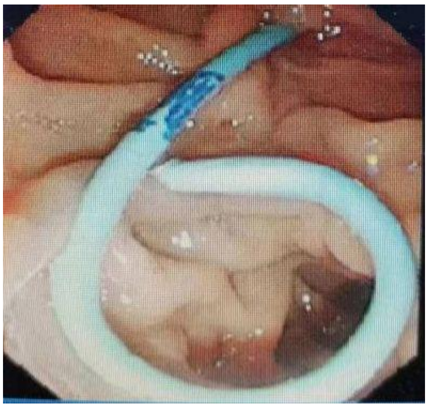

Figure 6: Results that showed matching made the factors more consistent between groups

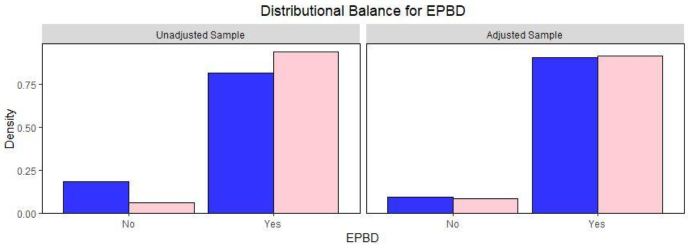

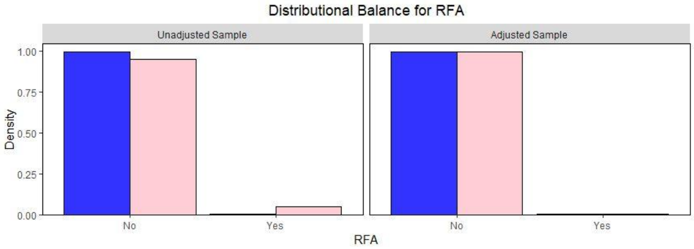

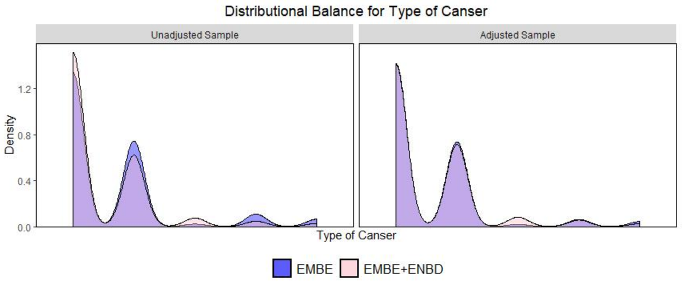
Distributional Balance for Type of Canser
Unadjusted Sample
Adjusted Sample
Density
Type of Canser
EMBE
EMBE+ENBD

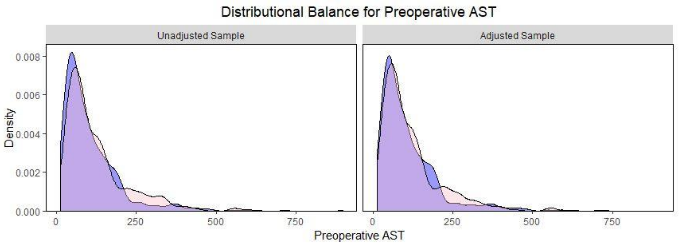
Distributional Balance for Preoperative AST
Unadjusted Sample
Adjusted Sample
Density
Preoperative AST

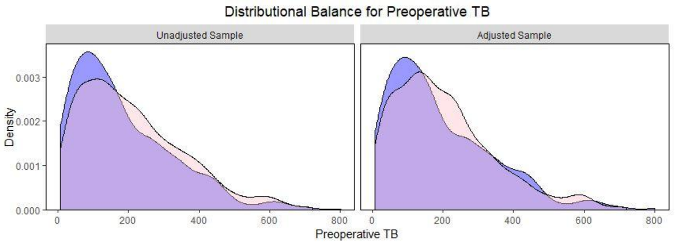
Distributional Balance for Preoperative TB
Unadjusted Sample
Adjusted Sample
Density
Preoperative TB

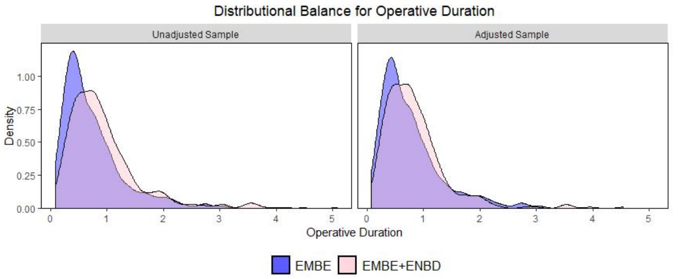
Distributional Balance for Operative Duration
Unadjusted Sample
Adjusted Sample
Density
Operative Duration
EMBE
EMBE+ENBD
